# Regeneration strategies after the adult mammalian central nervous system injury—biomaterials

**DOI:** 10.1093/rb/rbw004

**Published:** 2016-03-08

**Authors:** Yudan Gao, Zhaoyang Yang, Xiaoguang Li

**Affiliations:** ^1^Department of Neurobiology, School of Basic Medical Sciences, Capital Medical University, Beijing 100069, China,; ^2^Department of Biomedical Engineering, School of Biological Science and Medical Engineering, Beihang University, Beijing 100191, China

**Keywords:** central nervous system injury, neurogenesis, biomaterials, axonal regeneration, neural stem/precursor cell

## Abstract

The central nervous system (CNS) has very restricted intrinsic regeneration ability under the injury or disease condition. Innovative repair strategies, therefore, are urgently needed to facilitate tissue regeneration and functional recovery. The published tissue repair/regeneration strategies, such as cell and/or drug delivery, has been demonstrated to have some therapeutic effects on experimental animal models, but can hardly find clinical applications due to such methods as the extremely low survival rate of transplanted cells, difficulty in integrating with the host or restriction of blood–brain barriers to administration patterns. Using biomaterials can not only increase the survival rate of grafts and their integration with the host in the injured CNS area, but also sustainably deliver bioproducts to the local injured area, thus improving the microenvironment in that area. This review mainly introduces the advances of various strategies concerning facilitating CNS regeneration.

## Introduction

The central nervous system (CNS) diseases, such as Parkinson’s disease, Alzheimer’s disease [1, 2] and traumas, are all caused by neuronal loss or injury, which lead to the sensory, locomotion and cognitive dysfunction because of the absence of the axonal growth stimulative factors, like local growth stimulative substances and extracellular matrix (ECM) protein, and the existence of axonal growth inhibitory factors, like myelin-associated proteins and the physical/chemical barriers formed by glial scars [3]. It has been widely accepted that there are neural stem/precursor cells (NSCs/NPCs) which can generate new neurons in multiple areas of the adult mammalian CNS, such as the olfactory bulb, hippocampus dentate gyrus, periventricular area and central canal of the spinal cord [4–9].

The adult neurogenesis is dually influenced by *in vivo* and *in vitro* environments. Under the normal condition, the stem/precursor cells in the above areas keep ‘silent’. While under stress or injury, they will be activated and then proliferate and differentiate mainly into glial fibrillary acidic protein (GFAP) positive astrocytes contributing to form scar tissue but almost no neurons [8–10]. This article focuses on the strategies about facilitating CNS regeneration, including cell transplantation and endogenous neurogenesis, especially using biomaterials to facilitate tissue regeneration and functional recovery after brain and spinal cord injury (SCI).

## The cell transplantation-based therapeutic strategy

Exogenous cells are transplanted after the CNS injury to substitute dead or injured tissues. This sounds attractive, but faces three problems: first, how to immobilize the transplanted cells at the injured local area to avoid their dispersion to other areas; second, cell survival and activity; third, integration with host tissues [11]. While injecting exogenous cells into the CNS injured area together with saline or media, cell aggregation is almost inevitable before the injection, consequently leading to a lowered cell activity; after the injection, cells migrate dispersively or in cluster to other tissue areas, which will be cleared up by immune cells and lose their biological functions ultimately. Meanwhile, the survival of transplanted cells is further restricted by the dreadful microenvironment and the absence of cell adhesion and survival factors at the injured area. Theoretically, the integration of transplanted cells with host tissues must be fast; actually, it is often hampered by physical and chemical barriers. To solve the aforementioned problems, researchers have tried to use biomaterials with good biocompatibility to facility the repair and regeneration.

**Figure 1.**
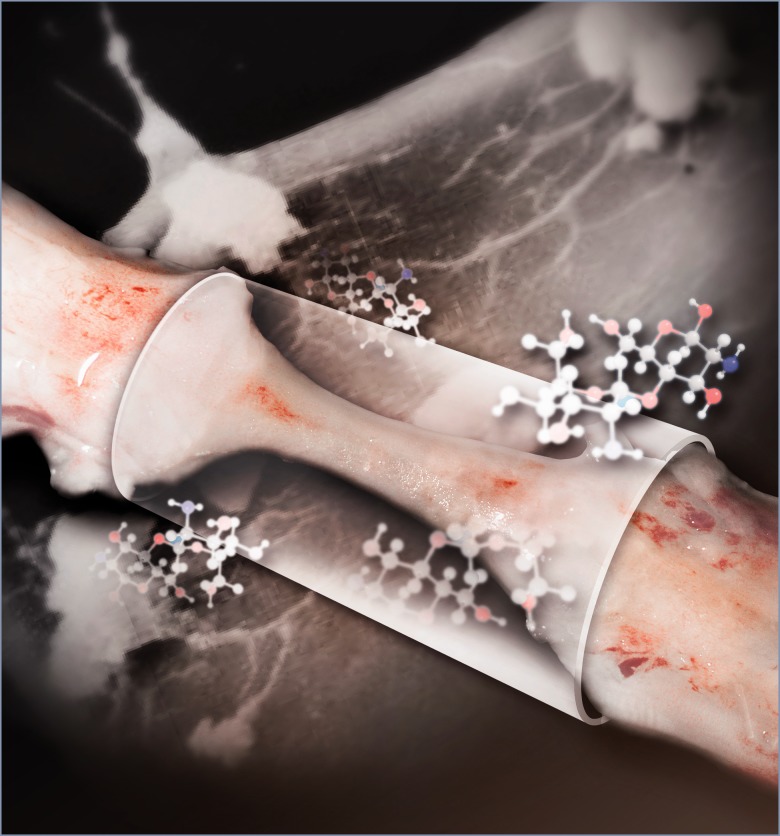
The regenerated nerve tissue inside an NT-3 loaded chitosan tube links the two ends of the completely transected and extracted rat spinal cord (5-mm gap) 6 months post operation. Good vascularization of the regenerated tissue is apparent. Chitosan molecular structures are shown. Inside the regenerated tissue, a large amount of newly generated neurons form a nascent neural network (shown here in the background), serving as relay stations to connect ascending and descending neural transmission signals to achieve sensory and motor functional recovery after spinal cord injury

## The drug/bioproduct-based therapeutic strategy

Bioactive molecules are delivered to the CNS to facilitate such tissue regeneration as neurogenesis, plasticity, axonal regeneration and neural protection function. For example, the injection or pumping of bioactive factors like epidermal growth factor (EGF) and erythropoietin into the brain ventricle can remarkably strengthen the activation and migration of endogenous NSCs/NPCs consequently leading to neurogenesis and improved functional recovery [12, 13]. Similarly, growth factors, such as interferon-g [14], glial cell line-derived neurotrophic factor (GDNF) [15] and neurotrophic factor-3 (NT-3), have been evidenced to play a certain role in protecting neurons and facilitating axonal regeneration after the SCI [16]. Unfortunately, the blood–brain barrier (BBB) and its low permeability restrict the diffusion via the routine delivery strategy in treatment [17]. A high general dosage, therefore, is needed to guarantee the therapeutic concentration at the injured local area, which often causes the cytotoxicity of the whole body. The general drug delivery may result in the target distribution of therapeutic molecules, together with some side effects, such as tumor formation and fibrillation [18]. We thus need to develop a new strategy to increase the BBB permeability of drugs, e.g., to deliver drugs through liposomes, NSCs/NPCs or biomaterials [16, 19, 20]. The direct drug delivery or transplantation bypassing the BBB, e.g., direct injection into the injured local site or intracerebroventricular (ICV) injection, may be taken into consideration, but probably accompanied by such risks as cerebral edema and convulsion, where drugs will diffuse quickly and get removed, with slight or no biological effects at all [21].

## The joint treatment strategy

The joint transplantation of cells, bioactive molecules and biomaterials enables the increased cell survival and integration, and realizes the local drug delivery in the brain, while bypassing the BBB and avoiding the general side effects [22, 23]. Similar to drug delivery, the intravenous injection of cells may potentially bring about the general side effects; meanwhile, it cannot directly enter or approach the injured local area [24]. Biomaterials may serve as the delivery carrier for such therapeutic molecules as growth factors, proteins and small molecules, providing a sustainable and adjustable drug release curve, with no requirement for multiple high-dosage treatments [16, 25]. They may also work as the tool for cell transportation and the scaffold for adhesion and migration, to make sure that the transplanted cells can stay at the injured local area and exert their functions.

## The biomaterial scaffold-based therapeutic strategy

Biomaterials, as a cell/drug delivery system, may offer concentrated and sustainable delivery. On the one hand, they may serve as the physical scaffold for cell adhesion, migration and growth; on the other hand, they may work as the carrier to combine with biomolecules and realize the oriented and sustainable delivery toward target sites. This avoids the surgical infection risk caused by multiple injections, as well as the easy diffusion and effect loss of soluble biomolecules.

Biomaterials scaffolds are generally considered to need surgery, Actually, according to the invasion degree, biomaterial scaffolds are categorized into the injectable and implantable scaffolds [26].

### Injectable scaffolds

In situ forming gel is a flowable liquid or sol pre-injection; once injected under the physiological environment, it forms gel quickly [27, 28] via the temperature variation, ion exchange process and light excitation [29–31]. For instance, collagen (Coll), methylcellulose (MC) and agarose are all temperature-sensitive polymers. Both Coll and MC form gel under the physiological condition; however, MC forms gel very slowly, which can be speeded up by mixing MC and hyaluronic acid (HAs) [32]. Although agarose needs a gel forming temperature lower than the physiological temperature, a freezing system is needed for its *in situ* formation [29]. Chitosan can be mixed with the guanosine 5′-diphosphate (GDP) solution and then quickly forms gel via the ion exchange process. To avoid the gel formation inside the injector, Chitosan solution and GDP solution must be separately injected with a binocular injector with two independent outlets [33].

The injectable scaffold has many advantages. It provides supporting as a biomaterial scaffold, while lowering the invasion to the minimum. However, its limitations in fast gel formation, high mechanical feature and biocompatibility hamper its abroad application [34].

### Implantable scaffolds

Except for the *in situ* forming gel, the other biomaterial scaffolds that need to be prepared before implantation to the injured area all belong to the implantable type. Compared with the injectable scaffold, the implantable scaffold has to be used under operation, thus accompanied by more severe invasion. Thanks to the scaffold pre-preparation before an implantation and various candidate methods for scaffold formation; the implantable scaffold has found wider applications.

The scaffolds used for CNS regeneration include morphologically noninjectable hydrogel/spongy scaffolds, with rich water content and multiporous structure to favor the cell adhesion and permeation [35]. The channel-type scaffolds in filamentous/mat/tubular structure, for instance, aim to reconstruct the axonal growth trajectory and direct neural regeneration [16, 36]. The nano-sized scaffolds, such as nanotube and nanofiber, tend to physically simulate the ECM and tubular structures, such as microtubules, axons and dendrites [22].

## Different-based biomaterials and their application in CNS regeneration

There are currently multiple strategies for repairing the CNS injury, e.g., to restrict inflammations and secondary injury, reconstruct the injured tissues, neutralize the disadvantageous molecules, strengthen the nutritive support and transplant exogenous cells [31–34], but all of which have limitations by single usage. Nowadays, the biomaterial scaffold–based research is drawing broad attention, which is expected to provide a supporting scaffold for neural regeneration, thus making it possible to create a local microenvironment favorable for regeneration; in the meantime, to combine with one or arbitrarily several strategies to develop a joint treatment protocol, ultimately facilitating the CNS regeneration. In this section, we introduce the biomaterial scaffold–based therapeutic strategy applied in CNS regeneration research in terms of biodegradable synthetic biomaterials and natural biomaterials.

### Biodegradable synthetic biomaterials

The synthetic biomaterials, unlike the natural materials originating from animals, do not need to face the great challenges of individual difference and disease propagation, with their synthesis processes and final components under relative control. As most synthetic materials do not have bioactivities, they have to be modified by grafting ECM polypeptides, growth factors or other bioactive factors to trigger neural regeneration. This part introduces the broadly applied polylactic acid (PLA)/polyglycolic acid (PGA)/poly(lactic-co-glycolic) acid (PLGA) and poly-epsilon-caprolactone (PCL).

#### *PGA*, *PLA and PLGA*

PGA, PLA and their copolymers have been widely applied in the field of tissue engineering due to their good biocompatibility and biodegradability. The PGA nanotube has gained the first approval from the U.S. Food and Drug Administration (U.S. FDA) to serve as the biodegradable synthetic peripheral nervous tube in clinics [35]. PLGA has also been approved by the FDA for clinical treatment in a wide range [37]. PGA, PLA and PLGA are well-known micron/nanoparticles for drug delivery, because their degradation speed can be controlled by adjusting the ratio of GA to LA; meanwhile, they can serve as the scaffold for *in situ* forming gel and slowly release NT-3 in a long term up to 2 weeks [22]. PLGA delivers Schwann cells, thus facilitating the neural regeneration in the SCI model of complete transection [38–40].

#### PCL

PCL is another polymer approved by the US FDA and widely applied in the CNS field. Compared with PLGA, PCL degrades at a slower speed and obtains a reduced acidity after degradation, consequently alleviating the inflammation reaction [41]. The PCL nanoline enables the cells adhering to the line to remain in the differentiating status for 7 days and form neural networks [42]. The modified PCL nanofiber scaffold, when carrying brain-derived neurotrophic factors (BDNF), can strengthen the proliferation of cortical stem cells and facilitate their differentiation into neurons and oligodendrocytes [43].

### Natural materials

#### Scaffolds composed of purified ECM components

ECM makes up about 20% of the whole CNS tissue in volume and plays a key role in maintaining cell functions [44]. The ECM in the peripheral tissue is rich in Coll, fibronectin (FN) and laminin (LN). The mature CNS ECM is mainly composed of glucosaminoglycan (HAs) and multiple proteoglycans [45]. In this part, we introduce the four ECM scaffolds of HA, Coll, FN and LN, as well as their joint treatment protocols applied in CNS regeneration.

HA is known to play roles in cellular processes like cell proliferation, morphogenesis, inflammation and wound repair. It interacts with cells via combining with CD44 and the surface receptors for hyaluronan-mediated motility [46, 47]. The HA alone cannot form gel and will quickly get degraded under enzyme effects. The hydrogel obtained by modifying HA with poly-lysine and Nogo-66 receptors is capable of increasing the neural fiber growth toward the injured area [48]. The injectable gel obtained by physically mixing HA and methyl cellulose is used to transfer growth factors to CNS [49–51]. The PLGA nanospheres with drugs further loaded and wrapped enable the prolonged drug release [52].

Coll-based biomaterials are prepared into filamentous and tubular structure in an attempt to reconnect the two ends of the injured area and direct the regenerate/sprout axonal trajectories [53, 54]. They are also cross-linked with genipin to form stable injectable gel for repairing SCI [55]. The Coll scaffold loaded with BDNF, when used in the thoracic semitransection model, enabled a longer axonal length growing toward the injured area as well as functional improvement [56]. After the traumatic brain injury (TBI), the Coll scaffold implanted with human marrow stromal cells (hMSCs) realized the decrease in lesion dimension, increase in spatial learning ability and functional recovery [57].

FN-based biomaterials are applied to CNS regeneration in the form of FN mat and injectable gel [58–60]. As the carrier for cell transplantation, the FN-based scaffold enables a more even distribution of NSCs in the injured brain area and a prolonged survival time up to 8 weeks [61]. The fibrin scaffold loaded with the NT-3 delivery system transplanted at 2 weeks after SCI could strengthen neural axon sprouting [62].

LN has been evidenced to be capable of facilitating the adhesion and migration of NSCs *in vitro*. It may also adjust the survival and proliferation of NSCs by the β1 integrin-mediated mechanism [63, 64]. According to published methods [61, 65, 66], LN and the polypeptides originating from LN are usually integrated with other biomaterials to increase their capabilities of supporting cell adhesion and survival, instead of direct application to CNS repair.

#### Decellularized intact ECM

Great progress in ECM-based bioscaffolds has been achieved in CNS repair, but the limitations of the relevant biomaterials are unnegligible. Even though those biomaterials provide the main ECM components, they cannot copy the morphology and structure of natural ECM. In research, only one type or two types of ECM proteins are adopted in scaffold preparation, while ECM is actually a complex of a series of proteins, growth factors and other cytokines. The research on decellularized intact ECM, therefore, has drawn broad attention.

The decellularized tissues originating from the peripheral nerve were first used in CNS regeneration research [67–70]. Then the decellularized muscle tissue was evidenced to offer effective matrixes to strengthen the axonal sprouting of spinal cord injured rats [71]. At present, the decellularized scaffold originating from the CNS is also used for studies on facilitating regeneration. The decellularization of brain and spinal cord neural tissues is carried out chemically [72] or by freezing and drying in combination with chemical methods [73, 74]. The decellularized scaffold possesses LN, FN, myelin and growth factors (vascular endothelial growth factor (VEGF) and fibroblast growth factor-2 (FGF-2)) [72, 73]. The decellularized CNS scaffold is capable of facilitating the proliferation, migration and differentiation of neural cells *in vitro* [72, 73], while avoiding the immune reaction when implanted into the body [72]. The decellularized scaffold may also be prepared as an injectable hydrogel and implanted via mini-invasive surgery [46, 75]. The urinary bladder matrix (U-ECM) obtained by decellularizing the bladder tissue was prepared as an injectable hydrogel and used for the rat TBI model, which enabled effective brain protection after the injury [76].

Although the ECM scaffolds maintain many bioactive factors, no published method is available to selectively remove growth inhibitory factors and retain growth promotive factors. Additionally, because of the complexity of various original tissue sources, a strict decellularization process is required to guarantee the complete removal of all cellular components, which might have side effects on host cells. Moreover, as ECM scaffolds are developed from mammalian tissues, individual differences between animals may have impact on the reconstruction results [77].

#### Other natural biomaterials

In the field of CNS regeneration, the widely used biomaterials also include agarose and chitosan [78–80]. Chitosan is a polysaccharide completely or partially deacetylated from chitin, and used in the CNS regeneration in the multiple forms of hydrogels, tubes, neural fibers and so on [81, 82]. Taking the spinal cord or brain injury of adult rats as the experimental models, our research team has long been devoted to using chitosan biomaterial scaffolds to repair CNS injury, and evidenced that the NT-3 chitosan scaffold enables the neural regeneration of the brain and spinal cord [20, 36, 83, 84], based on which we have further revealed that the NT-3 chitosan scaffold can activate endogenous neurogenesis and thus realize the functional recovery after rat SCI [16], as well as explored its underlying molecular mechanism via transcriptome analysis [85]. Our team reported on the first activation of endogenous neurogenesis via biomaterials in the world, and this is of significant value in the field of CNS regeneration. The bioactive scaffold developed by our team has been approved by China FDA and is now under the clinical trial.

## Endogenous neurogenesis

Contrary to the previous opinion that adult brain neurons remain static and cannot regenerate, the adult neurons have been found to be capable of generating new neurons, which will then be integrated into the complex host neural circuit. A large number of NSCs/NPCs are observed in multiple areas such as the olfactory bulb, hippocampus dentate gyrus, periventricular zone and central canal of the spinal cord [4–6, 9, 10, 86, 87].

The discovery of endogenous NSCs has brought new hopes to repairing brain injury and SCI or disease via neural transplantation and cell substitution. The adult neurogenesis refers to the whole neuronal development event from the very beginning of the stem/precursor cell division to its maturation and integration, as well as the appearance of new functional neurons till the survival end. Sometimes stem/precursor cells proliferation is mistaken as neurogenesis [88].

## Functional neurogenesis in adult nonmammalian vertebrates

The adult neurogenesis has been observed in many nonmammalian vertebrates. The medial cerebral cortex of lizards is similar to the hippocampus dentate gyrus of mammalians, which has neurogenesis after birth and can regenerate when responding to injury [89]. A salamander can regenerate its tail, limbs, mouth and eyes as well as neurons at the corresponding sites [90]. The retinal neurogenesis happens in the whole life of goldfish [91]. More impressively, its retina can regenerate even after partial cutting and removal [92]. Although significant neural tissue regeneration has been revealed in nonmammalians, its value to mammalians remains unclear. As pointed out by some researchers, why mammalians have lost such functions may be attributed to the selective evolution pressure [93].

The brain complexity of birds is quite similar to that of mammalians, also with neurogenesis after birth [94, 95]. In songbirds, new neurons are constantly supplemented to the senior sounding center [96, 97], the brain area for tweeting learning [98] and other special brain areas (but not all the neurons).

## Neurogenesis in adult mammalians

Ramony Cajal once asserted that ‘In the adult CNS, the neural circuits is fixed in some degree, terminated and unchangeable. Each neuron will die but not regenerate’. As the CNS injury and neurodegeneration diseases will not recover naturally, and neurogenesis distribution is quite limited in the adult mammalian brain, the researchers of this field thus drew the conclusion that neurogenesis was impossible in the adult mammalian brain. Using the sensitive methods, Joseph Altman first detected the constant neuronal mitosis in the adult brain. The endogenous neurogenesis can be observed in the brain hippocampus [4] and olfactory bulb [5] by using thymidine as the mitosis marker.

Later, more and more studies suggest that, under normal conditions, neurogenesis is observed in the subventricular zone (SVZ), subgranula zone(SGZ), olfactory bulb and the central canal of the spinal cord of adult mammalians [4–10].

The transplantation research supports the classification of the neurogenesis area and non-neurogenesis area in the CNS, and has evidenced the impact of microenvironments on NSC/NPC potential. If stem/precursor cells are transplanted into the neurogenesis area, they will differentiate into specific neurons in an area-specific pattern [99, 100]. When transplanting the SVZ stem/precursor cells to the hippocampus, hippocampus neurons will be generated; when transplanting SGZ stem/precursor cells to the rostral migratory stream, olfactory interneurons will be generated [101]. When transplanting the above two types of stem/precursor cells into the non-neurogenesis area, only glial cells will be generated. Taken together, neurogenesis relies on the local microenvironment that permits neurogenesis, but not on stem/precursor cell types with different properties in different areas. The impact of local microenvironments on stem/precursor cell behavior and its potential to differentiate into neurons indicates that it is of great importance to explore the molecular control mechanism of different-typed stem/precursor cells differentiation in the adult CNS.

The neurogenesis area has been redefined based on the neurogenesis permission by local microenvironments, instead of the existing sites of NSCs/NPCs, and this astonishes many researchers in this field. NSCs/NPCs have been discovered in many brain areas, including the white matter tract [102, 103], and may exist in the whole brain, although at a very low density [104]. These broadly distributed NSCs/NPCs seem to have no essential difference, although they are distributed unevenly, with significantly different growth dynamics and differentiation potentials. Their functions beyond the traditional neurogenesis areas remain unknown, not even their relationship with the stem/precursor cells inside the neurogenesis areas. For example, the NSCs/NPCs originating from the spinal cord are quite similar to those from the SGZ and SVZ *in vitro* [105]. When they are transplanted into the hippocampus, they gain the multipotent stem cell potential and generate granule neurons; when *in situ* or transplanted back to the original site of the spinal cord, they generate only glial cells but no neurons [100]. As revealed recently, neurogenesis can be facilitated by changing the microenvironment of the injured area after the adult rodent SCI, ultimately leading to the functional recovery of paralytic limbs [16, 85]. In sum, the neurogenesis degree in a specific area is determined by the local microenvironment and the stem/precursor cells with neurogenesis potential.

We thus come to the conclusion that the neurogenesis areas and non-neurogenesis areas are conceptually different, which reflect the complicated molecular and functional mechanisms, but not the fixed cellular environment. Will it be possible to operate the non-neurogenesis area to trigger neurogenesis? Under some pathological conditions is it possible to induce the variation of neurogenesis potential? Although without any evidence up to now, it is inferred that such neurogenesis variation may be realized via changing local molecular microenvironment, which is similar to the situation observed during the neural system development and in the adult neurogenesis area.

Several research groups have recently shown that, when selective neuronal death or degeneration occurs, neurogenesis may be induced in some degree in the normal non-neurogenesis areas. Scientists have also found that, under normal conditions, when endogenous multipotent stem/precursor cells normally at the adult brain are transplanted to the new cortex without neurogenesis, they can be induced to differentiate into neurons. This result has already been extended to corticospinal motor neurons [106].

Scientists have attempted to operate endogenous stem/precursor cells to repair the brain injury or SCI. The ICV injection of EGF or transforming growth factor α significantly increased proliferation of SVZ precursor cells, and the injection of FGF-2 slightly increased such proliferation [107, 108]. Even the subcutraneous injection of FGF-2 could also induce the proliferation of SVZ precursor cells [109]. Although the mitosis-induced nascent cells are distributed in the brain areas surrounding the brain ventricle, however, they usually cannot differentiate into neurons [108]. Using the *in vivo* local ischemic models, Nakatomi *et al.* showed that, after the injury and degradation in the CA1 area, the pumping of high-level EGF and FGF-2 enabled the neuronal regeneration in this area [12], and the regenerated neurons originated from the NSCs proliferation reaction in the back areas surrounding the brain ventricle. In spite of the high levels of EGF and FGF-2 largely exceeding the reasonable dosage for human application, these experiments did strengthen the endogenous neurogenesis reaction. In the field of repairing adult SCI, the team led by Li made use of the anti-inflammation feature of chitosan and the NT-3 slow release technique to improve the local microenvironment of the injured spinal cord area, activate spinal cord endogenous NSCs and induce them to migrate into the injured area, differentiate into neurons and establish contact with host neurons [16, 85]. Taken together, the operation on microenvironments seemingly supports and directs endogenous neurogenesis.

Other cytokines may also serve as important regulatory factors for neurogenesis, such as Noggin [110], VEGF [111] and BDNF [112]. The ICV injection of BDNF could increase the number of nascent neurons in the olfactory bulb of adult animals [113]. Further research has showed that the ICV administration of BDNF not only strengthens the proliferation of SVZ precursor cells, but also facilitates the neuronal migration to other areas, like the neostriatum, phren area, thalamencephalon and hypothalamencephalon [112, 114]. These results indicate that the utilization of growth factors inside the adult body possibly has impact on the fate of *in vivo* endogenous NSCs/NPCs, resulting in the substitution of the lost neurons caused by disease, degradation or death in the brain area. However, the feasibility and safety of these methods are still under debate. For example, it was reported that the ICV injection of EGF might lead to a large area hyperplasy of the brain ventricle wall [108].

Besides growth factors and neurotrophic factors, many other molecular and extracellular control patterns have been found to have potential effects on the behavior of SVZ NSCs/NPCs. For example, transcription factor E2F1 [115] and homebox gene *Vax1* [116] participate in the adjustment of adult SVZ neurogenesis. Additionally, messenger RNA (mRNA)-binding proteins Musashi1 [117], CCg [118] and orphan receptor TLX [119] are also involved in the adjustment of SVZ NSCs/NPCs proliferation and differentiation.

Moreover, researchers have demonstrated that doing exercises may also increase neurogenesis [120]. On the contrary, stress may decrease the neurogenesis in rodents [121] and primates [122], and the inflammation caused by X radiation also reduces neurogenesis [123]. In conclusion, the above candidate methods have to be repeatedly verified in nonhuman primate animals before the clinical trial, to optimize their safety and efficacy. Only under this prerequisite may the endogenous stem/precursor cell operation-based neuron substitution therapy be realized in future.

## Conclusions on endogenous CNS neurogenesis and its future prospects

A better understanding of the cellular and molecular control mechanism of NSCs/NPCs differentiation during the developmental stage and in the adult CNS is of great significance to activating endogenous neurogenesis and reconstructing the functional neural circuit lost because of injuries or diseases. In the adult mammalian brain, endogenous stem/precursor cells would be directed to develop and integrate, and to substitute the lost neurons. This application prospect is exciting, toward which great advances have been achieved, including: neurogenesis constantly occurs in multiple areas of the adult mammalian brain, the limited neurogenesis in the non-neurogenesis areas may be activated under proper conditions. The molecular/genetic control of lineage-specific differentiation is pushing forward the relevant research toward the goal of cell regeneration and repair.

In fact, many problems have to be solved before the realization of neuron substitution therapy using endogenous stem/precursor cells. First, we need to explore multiple signals responsible for the division, migration, differentiation and axonal growth. Of note, the potential therapy of *in situ* operating endogenous stem/precursor cells may not be limited to the brain area close to the adult neurogenesis area. In terms of safety in clinics, more attention should be paid to the research on endogenous stem cells activation.

In the future, brain and spinal cord repair may be realized by specifically activating endogenous NSCs/NPCs, which will then differentiate along the lineage of needed neuron cells to induce cell regeneration in the lesioned or diseased brain and spinal cord. The future studies should be focused on exploring the NSC/NPC potentials in different local microenvironments, as well as the complex interaction between signals. Special attention should also be paid to the nonuniformity of stem/precursor cells and how to specifically adjust the developmental signals of NSCs, as well as neuronal differentiation and survival making use of the cell type limitations, environmental permission and direction. In the coming 10 years, endogenous neurogenesis will revolutionarily push forward the advancement of nervous repair research.

## References

[1] Citron M. Alzheimer’s disease: strategies for disease modification. Nat Rev Drug Discov 2010;9:387–98. 20431570 10.1038/nrd2896

[2] Lassmann H Horssen JV Mahad D. Progressive multiple sclerosis: pathology and pathogenesis. Nat Rev Neurol 2012;8:647–56. 23007702 10.1038/nrneurol.2012.168

[3] Fitch MT Silver J. CNS injury, glial scars, and inflammation: inhibitory extracellular matrices and regeneration failure. Exp Neurol 2008;209:294–301. 17617407 10.1016/j.expneurol.2007.05.014 PMC2268907

[4] Altman J Das GD. Autoradiographic and histological evidence of postnatal hippocampal neurogenesis in rats. J Comp Neurol 1965;124:319–35. 5861717 10.1002/cne.901240303

[5] Altman J. Autoradiographic and histological studies of postnatal neurogenesis. IV. Cell proliferation and migration in the anterior forebrain, with special reference to persisting neurogenesis in the olfactory bulb. J Comp Neurol 1969;137:433–57. 5361244 10.1002/cne.901370404

[6] Reynolds BA Weiss S. Generation of neurons and astrocytes from isolated cells of the adult mammalian central nervous system. Science 1992;255:1707–10. 1553558 10.1126/science.1553558

[7] Richards LJ Kilpatrick TJ Bartlett PF. De novo generation of neuronal cells from the adult mouse brain. Proc Natl Acad Sci U S A 1992;89:8591–5. 1528866 10.1073/pnas.89.18.8591 PMC49966

[8] Mothe AJ Tator CH. Proliferation, migration, and differentiation of endogenous ependymal region stem/progenitor cells following minimal spinal cord injury in the adult rat. Neuroscience 2005;131:177–87. 15680701 10.1016/j.neuroscience.2004.10.011

[9] Barnabé-Heider F Göritz C Sabelström H. Origin of new glial cells in intact and injured adult spinal cord. Cell Stem Cell 2010;7:470–82. 20887953 10.1016/j.stem.2010.07.014

[10] Gage FH. Mammalian neural stem cells. Science 2000;287:1433–8. 10688783 10.1126/science.287.5457.1433

[11] Roger YT Tobias F Nikolaos M. Regenerative therapies for central nervous system diseases: a biomaterials approach. Neuropsychopharmacology 2014;39:169–88. 24002187 10.1038/npp.2013.237 PMC3857664

[12] Hirofumi N Toshihiko K Shigeo O. Regeneration of hippocampal pyramidal neurons after ischemic brain injury by recruitment of endogenous neural progenitors. Cell 2002;110:429–41. 12202033 10.1016/s0092-8674(02)00862-0

[13] Kolb B Morshead C Gonzalez C. Growth factor-stimulated generation of new cortical tissue and functional recovery after stroke damage to the motor cortex of rats. J Cereb Blood Flow Metab 2007;27:983–97. 16985505 10.1038/sj.jcbfm.9600402

[14] Victorio SCS Havton LA Oliveira ALR. Absence of IFN gamma expression induces neuronal degeneration in the spinal cord of adult mice. J Neuroinflamm 2010;7:77–90. 10.1186/1742-2094-7-77 PMC2993684 21073708

[15] Zhang LQ Ma ZW Smith GM. GDNF-enhanced axonal regeneration and myelination following spinal cord injury is mediated by primary effects on neurons. Glia 2009;57:1178–91. 19170182 10.1002/glia.20840 PMC2855953

[16] Yang Z Zhang AF Duan HM. NT-3-chitosan elicits robust endogenous neurogenesis to enable functional recovery after spinal cord injury. Proc Natl Acad Sci U S A 2015;112:13354–9. 26460015 10.1073/pnas.1510194112 PMC4629318

[17] Pardridge WM. Drug transport across the blood-brain barrier. J Cereb Blood Flow Metab 2012;32:1959–72. 22929442 10.1038/jcbfm.2012.126 PMC3494002

[18] Lee RJ Springer ML Blanco-Bose WE. VEGF gene delivery to myocardium: deleterious effects of unregulated expression. Circulation 2000;102:898–901. 10952959 10.1161/01.cir.102.8.898

[19] Patel MM Goyal BR Bhadada SV. Getting into the brain: approaches to enhance brain drug delivery. CNS Drugs 2009;23:35–58. 19062774 10.2165/0023210-200923010-00003

[20] Mo LH Yang ZY Zhang AF Li XG. The repair of the injured adult rat hippocampus with NT-3-chitosan carriers. Biomaterials 2010;31:2184–92. 20022104 10.1016/j.biomaterials.2009.11.078

[21] Groothuis DR. The blood-brain and blood-tumor barriers: a review of strategies for increasing drug delivery. Neuro Oncol 2000;2:45–59. 11302254 10.1093/neuonc/2.1.45 PMC1920694

[22] Orive G Anitua E Pedraz JL. Biomaterials for promoting brain protection, repair and regeneration. Nat Rev Neurosci 2009;10:682–92. 19654582 10.1038/nrn2685

[23] Pakulska MM Ballios BG Shoichet MS. Injectable hydrogels for central nervous system therapy. Biomed Mater 2012;7:024101 22456684 10.1088/1748-6041/7/2/024101

[24] Quertainmont R Cantinieaux D Botman O. Mesenchymal stem cell graft improves recovery after spinal cord injury in adult rats through neurotrophic and pro-angiogenic actions. PLoS One 2012;7:e39500 22745769 10.1371/journal.pone.0039500 PMC3380009

[25] Hoare TR Kohane DS. Hydrogels in drug delivery: progress and challenges. Polymer 2008;49:1993–2007.

[26] Zhong Y Bellamkonda RV. Biomaterials for the central nervous system. J R Soc Interface 2008;5:957–75. 18477539 10.1098/rsif.2008.0071 PMC2475552

[27] Yu L Ding J. Injectable hydrogels as unique biomedical materials. Chem Soc Rev 2008;37:1473–81. 18648673 10.1039/b713009k

[28] Jindřich K. Hydrogel biomaterials: a smart future? Biomaterials 2007;28:5185–92. 17697712 10.1016/j.biomaterials.2007.07.044 PMC2212614

[29] Jain A Kim Y Mckeon R. In situ gelling hydrogels for conformal repair of spinal cord defects, and local delivery of BDNF after spinal cord injury. Biomaterials 2006;27:497–504. 16099038 10.1016/j.biomaterials.2005.07.008

[30] Lin HR Sung KC Vong WJ. In situ gelling of alginate/pluronic solutions for ophthalmic delivery of pilocarpine. Biomacromolecules 2004;5:2358–65. 15530052 10.1021/bm0496965

[31] Piantino J Burdick JA Goldberg D. An injectable, biodegradable hydrogel for trophic factor delivery enhances axonal rewiring and improves performance after spinal cord injury. Exp Neurol. 2006;201:359–67. 16764857 10.1016/j.expneurol.2006.04.020

[32] Gupta D Tator CH Shoichet MS. Fast-gelling injectable blend of hyaluronan and methylcellulose for intrathecal, localized delivery to the injured spinal cord. Biomaterials 2006;27:2370–9. 16325904 10.1016/j.biomaterials.2005.11.015

[33] Mekhail M Daoud J Almazan Rapid, guanosine 5′-diphosphate-induced, gelation of chitosan sponges as novel injectable scaffolds for soft tissue engineering and drug delivery applications. Adv Healthc Mater 2013;2:1126–30. 23554366 10.1002/adhm.201200371

[34] Emily H Anthony L Michele M. Synthesis and characterization of an injectable hydrogel with tunable mechanical properties for soft tissue repair. Biomacromolecules 2006;7:3223–8. 17096554 10.1021/bm0602536

[35] Assunção-Silva RC Gomes ED Sousa N. Hydrogels and cell based therapies in spinal cord injury regeneration. Stem Cells Int 2015;2015:1–24. 10.1155/2015/948040 PMC4466497 26124844

[36] Li XG Yang ZY Zhang AF. Repair of thoracic spinal cord injury by chitosan tube implantation in adult rats. Biomaterials 2009;30:1121–32. 19042014 10.1016/j.biomaterials.2008.10.063

[37] Thompson DM Buettner HM. Neurite outgrowth is directed by Schwann cell alignment in the absence of other guidance cues. Ann Biomed Eng 2006;34:161–8. 16453203 10.1007/s10439-005-9013-4

[38] Chen BK Knight AM Madigan NN. Comparison of polymer scaffolds in rat spinal cord: a step toward quantitative assessment of combinatorial approaches to spinal cord repair. Biomaterials 2011; 32:8077–86. 21803415 10.1016/j.biomaterials.2011.07.029 PMC3163757

[39] Moore MJ Friedman JA Lewellyn EB. Multiple-channel scaffolds to promote spinal cord axon regeneration. Biomaterials 2006;27:419–29. 16137759 10.1016/j.biomaterials.2005.07.045

[40] Olson HE Rooney GE Louann G. Neural stem cell- and Schwann cell-loaded biodegradable polymer scaffolds support axonal regeneration in the transected spinal cord. Tissue Eng Part A 2009;15:1797–805. 19191513 10.1089/ten.tea.2008.0364 PMC2792101

[41] Wong DY Hollister SJ Krebsbach PH. Poly(epsilon-caprolactone) and poly (L-lactic-co-glycolic acid) degradable polymer sponges attenuate astrocyte response and lesion growth in acute traumatic brain injury. Tissue Eng 2007;13:2515–23. 17655492 10.1089/ten.2006.0440

[42] Horne MK Nisbet DR Forsythe JS. Three-dimensional nanofibrous scaffolds incorporating immobilized BDNF promote proliferation and differentiation of cortical neural stem cells. Stem Cells Dev 2010;19:843–52. 19831634 10.1089/scd.2009.0158

[43] Bechara SL Judson A Popat KC. Template synthesized poly(ε-caprolactone) nanowire surfaces for neural tissue engineering. Biomaterials 2010;31:3492–501. 20149440 10.1016/j.biomaterials.2010.01.084

[44] Sobel RA. The extracellular matrix in multiple sclerosis lesions. J Neuropathol Exp Neurol 1998;57:205–17. 9600212 10.1097/00005072-199803000-00001

[45] Dequach JA Yuan SH Goldstein LS. Decellularized porcine brain matrix for cell culture and tissue engineering scaffolds. Tissue Eng Part A 2011;17:2583–92. 21883047 10.1089/ten.tea.2010.0724 PMC3204197

[46] Sahoo S Khetan CS. Hydrolytically degradable hyaluronic acid hydrogels with controlled temporal structures. Biomacromolecules 2008;9:1088–92. 18324776 10.1021/bm800051m PMC2667229

[47] Hall CL Turley EA. Hyaluronan: RHAMM mediated cell locomotion and signaling in tumorigenesis. J Neurooncol 1995;26:221–9. 8750188 10.1007/BF01052625

[48] Wei Y He Y Xu C. Hyaluronic acid hydrogel modified with nogo-66 receptor antibody and poly-L-lysine to promote axon regrowth after spinal cord injury. J Biomed Mater Res B Appl Biomater 2010;95b:110–7. 20725955 10.1002/jbm.b.31689

[49] Kang CE Poon PC Tator CH. A new paradigm for local and sustained release of therapeutic molecules to the injured spinal cord for neuroprotection and tissue repair. Tissue Eng Part A 2008;15:595–604. 18991489 10.1089/ten.tea.2007.0349

[50] Cooke MJ Wang Y Morshead CM. Controlled epi-cortical delivery of epidermal growth factor for the stimulation of endogenous neural stem cell proliferation in stroke-injured brain. Biomaterials 2011;32:5688–97. 21550655 10.1016/j.biomaterials.2011.04.032

[51] Kang CE Tator CH Shoichet MS. Poly(ethylene glycol) modification enhances penetration of fibroblast growth factor 2 to injured spinal cord tissue from an intrathecal delivery system. J Control Release 2010;144:25–31. 20114065 10.1016/j.jconrel.2010.01.029

[52] Baumann MD Kang CE Stanwick JC. An injectable drug delivery platform for sustained combination therapy. J Control Release 2009;138:205–13. 19442692 10.1016/j.jconrel.2009.05.009

[53] Liu T Houle JD Xu J. Nanofibrous collagen nerve conduits for spinal cord repair. Tissue Eng Part A 2012;18:1057–66. 22220714 10.1089/ten.tea.2011.0430 PMC3338103

[54] Yoshii S Ito S Shima M. Functional restoration of rabbit spinal cord using collagen-filament scaffold. J Tissue Eng Regen Med 2009;3:19–25. 19012267 10.1002/term.130

[55] Macaya D Ng KK Spector M. Injectable collagen–genipin gel for the treatment of spinal cord injury: in vitro studies. Adv Funct Mater 2011;21:4788–97.

[56] Qianqian H Wenjie S Hang L. Linear ordered collagen scaffolds loaded with collagen-binding brain-derived neurotrophic factor improve the recovery of spinal cord injury in rats. Tissue Eng Part A 2009;15:2927–35. 19290803 10.1089/ten.TEA.2008.0506

[57] Dunyue L Asim M Changsheng Q. Collagen scaffolds populated with human marrow stromal cells reduce lesion volume and improve functional outcome after traumatic brain injury. Neurosurgery 2007;61:596–602. 17881974 10.1227/01.NEU.0000290908.38438.B2 PMC1994819

[58] King VR Alla A Wei DYT. The use of injectable forms of fibrin and fibronectin to support axonal ingrowth after spinal cord injury. Biomaterials 2010;31:4447–56. 20206381 10.1016/j.biomaterials.2010.02.018

[59] King VR Hewazy D Alovskaya A. The neuroprotective effects of fibronectin mats and fibronectin peptides following spinal cord injury in the rat. Neuroscience 2010;168:523–30. 20347014 10.1016/j.neuroscience.2010.03.040

[60] Phillips JB King VR Ward Z. Fluid shear in viscous fibronectin gels allows aggregation of fibrous materials for CNS tissue engineering. Biomaterials 2004;25:2769–79. 14962555 10.1016/j.biomaterials.2003.09.052

[61] Tate CC Shear DA Tate MC. Laminin and fibronectin scaffolds enhance neural stem cell transplantation into the injured brain. J Tissue Eng Regen Med 2009;3:208–17. 19229887 10.1002/term.154

[62] Johnson PJ Parker SR Sakiyama-Elbert SE. Controlled release of neurotrophin-3 from fibrin-based tissue engineering scaffolds enhances neural fiber sprouting following subacute spinal cord injury. Biotechnol Bioeng 2009;104:1207–14. 19603426 10.1002/bit.22476 PMC2780336

[63] Tate MC Garcı´ AAJ Keselowsky BG. Specific beta1 integrins mediate adhesion, migration, and differentiation of neural progenitors derived from the embryonic striatum. Mol Cell Neurosci 2004;27:22–31. 15345240 10.1016/j.mcn.2004.05.001

[64] Leone DP Relvas JB Campos LS. Regulation of neural progenitor proliferation and survival by β1 integrins. J Cell Sci. 2005;118:2589–99. 15928047 10.1242/jcs.02396

[65] Green RA Lovell NH Poole-Warren LA. Impact of co-incorporating laminin peptide dopants and neurotrophic growth factors on conducting polymer properties. Acta Biomater 2010;6:63–71. 19563922 10.1016/j.actbio.2009.06.030

[66] Nakaji-Hirabayashi T Kato K Iwata H. Improvement of neural stem cell survival in collagen hydrogels by incorporating laminin-derived cell adhesive polypeptides. Bioconjug Chem 2012;23:212–21. 22229651 10.1021/bc200481v

[67] Berry M Hall S Follows R. Response of axons and glia at the site of anastomosis between the optic nerve and cellular or acellular sciatic nerve grafts. J Neurocytol 1988;17:727–44. 3148025 10.1007/BF01216702

[68] Berry M Rees L Hall S. Optic axons regenerate into sciatic nerve isografts only in the presence of Schwann cells. Brain Res Bull 1988;20:223–31. 3370505 10.1016/0361-9230(88)90182-7

[69] Dezawa M Nagano T. Contacts between regenerating axons and the Schwann cells of sciatic nerve segments grafted to the optic nerve of adult rats. J Neurocytol 1993;22:1103–12. 8106882 10.1007/BF01235752

[70] Smith GV Stevenson JA. Peripheral nerve grafts lacking viable Schwann cells fail to support central nervous system axonal regeneration. Exp Brain Res 1988;69:299–306. 3278916 10.1007/BF00247575

[71] Zhang XY Xue H Liu JM. Chemically extracted acellular muscle: a new potential scaffold for spinal cord injury repair. J Biomed Mater Res A 2012;100:578–87. 22213649 10.1002/jbm.a.33237

[72] Guo SZ Ren XJ Wu B. Preparation of the acellular scaffold of the spinal cord and the study of biocompatibility. Spinal Cord 2010;48:576–81. 20065987 10.1038/sc.2009.170

[73] Crapo PM Medberry CJ Reing JE. Biologic scaffolds composed of central nervous system extracellular matrix. Biomaterials 2012;33:3539–47. 22341938 10.1016/j.biomaterials.2012.01.044 PMC3516286

[74] Domenico R Maria Teresa C Beatrice N. Angiogenic response induced by acellular brain scaffolds grafted onto the chick embryo chorioallantoic membrane. Brain Res 2003;989:9–15. 14519506 10.1016/s0006-8993(03)03225-6

[75] Medberry CJ Crapo PM Siu BF. Hydrogels derived from central nervous system extracellular matrix. Biomaterials 2013;34:1033–40. 23158935 10.1016/j.biomaterials.2012.10.062 PMC3512573

[76] Wang JY Liou AKF Ren ZH. Neurorestorative effect of urinary bladder matrix-mediated neural stem cell transplantation following traumatic brain injury in rats. CNS Neurol Disord Drug Targets 2013;12:413–25. 23469853 10.2174/1871527311312030014 PMC4049096

[77] Meng F Modo M Badylak SF. Biologic scaffold for CNS repair. Regen Med 2014;9:367–83. 24935046 10.2217/rme.14.9

[78] Lynam DA Shahriari D Wolf KJ. Brain derived neurotrophic factor release from layer-by-layer coated agarose nerve guidance scaffolds. Acta Biomater 2015;18:128–31. 25712385 10.1016/j.actbio.2015.02.014

[79] Gao M Lu P Bednark B. Templated agarose scaffolds for the support of motor axon regeneration into sites of complete spinal cord transection. Biomaterials 2013;34:1529–36. 23182350 10.1016/j.biomaterials.2012.10.070 PMC3518618

[80] Martin BC Minner EJ Wiseman SL. Agarose and methylcellulose hydrogel blends for nerve regeneration applications. J Neural Eng 2008;5:221–31. 18503105 10.1088/1741-2560/5/2/013

[81] Hiroshi N Tasneem Z Howard K. Extramedullary chitosan channels promote survival of transplanted neural stem and progenitor cells and create a tissue bridge after complete spinal cord transection. Tissue Eng Part A 2008;14:649–65. 18419246 10.1089/tea.2007.0180

[82] Gnavi S Barwig C Freier T. The use of chitosan-based scaffolds to enhance regeneration in the nervous Ssstem. Int Rev Neurobiol 2013;109:1–62. 24093605 10.1016/B978-0-12-420045-6.00001-8

[83] Li XG Yang ZY Zhang AF. The effect of neurotrophin-3/chitosan carriers on the proliferation and differentiation of neural stem cells. Biomaterials 2009;30:4978–85. 19539985 10.1016/j.biomaterials.2009.05.047

[84] Yang ZY Duan HM Mo LH. The effect of the dosage of NT-3/chitosan carriers on the proliferation and differentiation of neural stem cells. Biomaterials 2010;31:4846–54. 20346501 10.1016/j.biomaterials.2010.02.015

[85] Duan HM Ge WH Zhang AF. Transcriptome analyses reveal molecular mechanisms underlying functional recovery after spinal cord injury. Proc Natl Acad Sci U S A 2015;112:13360–5. 26460053 10.1073/pnas.1510176112 PMC4629389

[86] Lois C Alvarez-Buylla A. Proliferating subventricular zone cells in the adult mammalian forebrain can differentiate into neurons and glia. Proc Natl Acad Sci U S A 1993;90:2074–7. 8446631 10.1073/pnas.90.5.2074 PMC46023

[87] Kooy DVD Weiss S. Why stem cells? Science 2000;287:1439–41. 10688784 10.1126/science.287.5457.1439

[88] Jason GE Bartley DM Sanjay SPM. The repair of complex neuronal circuitry by transplanted and endogenous precursors. NeuroRx 2004;1:452–71. 15717047 10.1602/neurorx.1.4.452 PMC534952

[89] Lopez-Garcia C Molowny A Martinez-Guijarro FJ. Lesion and regeneration in the medial cerebral cortex of lizards. Histol Histopathol 1992;7:725–46. 1457995

[90] Brockes JP. Amphibian limb regeneration: rebuilding a complex structure. Science 1997;276:81–7. 9082990 10.1126/science.276.5309.81

[91] Johns PR Easter SS. Growth of the adult goldfish eye. II. Increase in retinal cell number. J Comp Neurol 1977;176:331–41. 915041 10.1002/cne.901760303

[92] Hitchcock PF Lindsey Myhr KJ Easter SS. Local regeneration in the retina of the goldfish. J Neurobiol 1992;23:187–203. 1527527 10.1002/neu.480230209

[93] Gotz M Hartfuss E Malatesta P. Radial glial cells as neuronal precursors: a new perspective on the correlation of morphology and lineage restriction in the developing cerebral cortex of mice. Brain Res Bull 2002;57:777–88. 12031274 10.1016/s0361-9230(01)00777-8

[94] Nottebohm F Alvarez-Buylla A Cynx J. Song learning in birds: the relation between perception and production. Philos Trans R Soc Lond B Biol Sci 1990;329:115–24. 1978358 10.1098/rstb.1990.0156

[95] Nottebohm F. Why are some neurons replaced in adult brain? J Neurosci 2002;22:624–8. 11826090 10.1523/JNEUROSCI.22-03-00624.2002 PMC6758507

[96] Goldman SA Nottebohm F. Neuronal production, migration, an differentiation in a vocal control nucleus of the adult female canary brain. Proc Natl Acad Sci U S A 1983;80:2390–4. 6572982 10.1073/pnas.80.8.2390 PMC393826

[97] Kirn JR Alvarez-Buylla A Nottebohm F. Production and survival of projection neurons in a forebrain vocal center of adult male canaries. J Neurosci 1991;11:1756–62. 2045885 10.1523/JNEUROSCI.11-06-01756.1991 PMC6575395

[98] Simpson HB Vicario DS. Brain pathways for learned and unlearned vocalizations differ in zebra finches. J Neurosci 1990;10:1541–56. 2332796 10.1523/JNEUROSCI.10-05-01541.1990 PMC6570078

[99] Gage FH Coates PW Palmer TD. Survival and differentiation of adult neuronal progenitor cells transplanted to the adult brain. Proc Natl Acad Sci U S A 1995;92:11879–83. 8524867 10.1073/pnas.92.25.11879 PMC40506

[100] Shihabuddin LS Horner PJ Ray J. Adult spinal cord stem cells generate neurons after transplantation in the adult dentate gyrus. J Neurosci 2000;20:8727–35. 11102479 10.1523/JNEUROSCI.20-23-08727.2000 PMC6773057

[101] Suhonen JO Peterson DA Ray J. Differentiation of adult hippocampus-derived progenitors into olfactory neurons in vivo. Nature 1996;383:624–7. 8857538 10.1038/383624a0

[102] Palmer TD Markakis EA Willhoite AR. Fibroblast growth factor-2 activates a latent neurogenic program in neural stem cells from diverse regions of the adult CNS. J Neurosci 1999;19:8487–97. 10493749 10.1523/JNEUROSCI.19-19-08487.1999 PMC6783019

[103] Kondo T Raff M. Oligodendrocyte precursor cells reprogrammed to become multipotential CNS stem cells. Science 2000;289:1754–7. 10976069 10.1126/science.289.5485.1754

[104] Belachew S Chittajallu R Aguirre AA. Postnatal NG2 proteoglycan-expressing progenitor cells are intrinsically multipotent and generate functional neurons. J Cell Biol 2003;161:169–86. 12682089 10.1083/jcb.200210110 PMC2172886

[105] Shihabuddin LS Ray J Gage FH. FGF-2 is sufficient to isolate progenitors found in the adult mammalian spinal cord. Exp Neurol 1997;148:577–86. 9417834 10.1006/exnr.1997.6697

[106] Chen J Magavi SS Macklis JD. Neurogenesis of corticospinal motor neurons extending spinal projections in adult mice. Proc Natl Acad Sci U S A 2004;101:16357–62. 15534207 10.1073/pnas.0406795101 PMC528970

[107] Craig CG Tropepe V Morshead CM. In vivo growth factor expansion of endogenous subependymal neural precursor cell populations in the adult mouse brain. J Neurosci 1996;16:2649–58. 8786441 10.1523/JNEUROSCI.16-08-02649.1996 PMC6578757

[108] Kuhn HG Winkler J Kempermann G. Epidermal growth factor and fibroblast growth factor-2 have different effects on neural progenitors in the adult rat brain. J Neurosci 1997;17:5820–9. 9221780 10.1523/JNEUROSCI.17-15-05820.1997 PMC6573198

[109] Tao Y Black IB DiCicco-Bloom E. Neurogenesis in neonatal rat brain is regulated by peripheral injection of basic fibroblast growth factor (bFGF). J Comp Neurol 1996;376:653–63. 8978476 10.1002/(SICI)1096-9861(19961223)376:4<653::AID-CNE11>3.0.CO;2-N

[110] Lim DA Tramontin AD Trevejo JM. Noggin antagonizes BMP signaling to create a niche for adult neurogenesis. Neuron 2000;28:713–26. 11163261 10.1016/s0896-6273(00)00148-3

[111] Jin K Zhu Y Sun Y. Vascular endothelial growth factor (VEGF) stimulates neurogenesis in vitro and in vivo. Proc Natl Acad Sci U S A 2002;99:11946–50. 12181492 10.1073/pnas.182296499 PMC129374

[112] Pencea V Bingaman KD Wiegand SJ. Infusion of brain-derived neurotrophic factor into the lateral ventricle of the adult rat leads to new neurons in the parenchyma of the striatum, septum, thalamus, and hypothalamus. J Neurosci 2001;21:6706–17. 11517260 10.1523/JNEUROSCI.21-17-06706.2001 PMC6763082

[113] Zigova T Pencea V Wiegand SJ. Intraventricular administration of BDNF increases the number of newly generated neurons in the adult olfactory bulb. Mol Cell Neurosci 1998;11:234–45. 9675054 10.1006/mcne.1998.0684

[114] Benraiss A Chmielnicki E Lerner K. Adenoviral brain-derived neurotrophic factor induces both neostriatal and olfactory neuronal recruitment from endogenous progenitor cells in the adult forebrain. J Neurosci 2001;21:6718–31. 11517261 10.1523/JNEUROSCI.21-17-06718.2001 PMC6763117

[115] Cooper-Kuhn CM Vroemen M Brown J. Impaired adult neurogenesis in mice lacking the transcription factor E2F1. Mol Cell Neurosci 2002;21:312–23. 12401450 10.1006/mcne.2002.1176

[116] Soria JM Taglialatela P Gil-Perotin S. Defective postnatal neurogenesis and disorganization of the rostral migratory stream in absence of the Vax1 homeobox gene. J Neurosci 2004;24:11171–81. 15590934 10.1523/JNEUROSCI.3248-04.2004 PMC6730283

[117] Sakakibara S Nakamura Y Satoh H. Rna-binding protein Musashi2: developmentally regulated expression in neural precursor cells and subpopulations of neurons in mammalian CNS. J Neurosci 2001;21:8091–107. 11588182 10.1523/JNEUROSCI.21-20-08091.2001 PMC6763847

[118] Taupin P Ray J Fischer WH. FGF-2-responsive neural stem cell proliferation requires CCg, a novel autocrine/paracrine cofactor. Neuron 2000;28:385–97. 11144350 10.1016/s0896-6273(00)00119-7

[119] Shi Y Chichung LD Taupin P. Expression and function of orphan nuclear receptor TLX in adult neural stem cells. Nature 2004;427:78–83. 14702088 10.1038/nature02211

[120] Gould E Reeves AJ Graziano MS. Neurogenesis in the neocortex of adult primates. Science 1999;286:548–52. 10521353 10.1126/science.286.5439.548

[121] Tanapat P Galea LA Gould E. Stress inhibits the proliferation of granule cell precursors in the developing dentate gyrus. Int J Dev Neurosci 1998;16:235–9. 9785120 10.1016/s0736-5748(98)00029-x

[122] Gould E Tanapat P McEwen BS. Proliferation of granule cell precursors in the dentate gyrus of adult monkeys is diminished by stress. Proc Natl Acad Sci U S A 1998;95:3168–71. 9501234 10.1073/pnas.95.6.3168 PMC19713

[123] Monje ML Toda H Palmer TD. Inflammatory blockade restores adult hippocampal neurogenesis. Science 2003;302:1760–5. 14615545 10.1126/science.1088417

